# Impact of obesity on outcome of severe bacterial infections

**DOI:** 10.1371/journal.pone.0251887

**Published:** 2021-05-19

**Authors:** Åsa Alsiö, Salmir Nasic, Lars Ljungström, Gunnar Jacobsson

**Affiliations:** 1 Research and Development Centre, Skaraborg Hospital, Skövde, Sweden; 2 Department of Infectious Diseases, Institute of Biomedicine, Sahlgrenska Academy at University of Gothenburg, Gothenburg, Sweden; 3 Department of Molecular and Clinical Medicine, Institute of Medicine, Sahlgrenska Academy at University of Gothenburg, Gothenburg, Sweden; 4 Department of Infectious Diseases, Skaraborg Hospital, Skövde, Sweden; 5 Center for Antibiotic Resistance Research (CARe), Department of Infectious Diseases, Institute of Biomedicine, Sahlgrenska Academy, University of Gothenburg, Gothenburg, Sweden; Heidelberg University Hospital, GERMANY

## Abstract

**Introduction:**

Obesity is a rapidly growing global health concern with considerable negative impact on life-time expectancy. It has yet not been clarified if and how obesity impacts outcomes of severe bacterial infections. The aim of this study was to determine how body mass index impacts outcome of severe bacterial infections in a well-defined population-based cohort.

**Methods:**

This study is based on a cohort of 2196 patients included in a Swedish prospective, population-based, consecutive observational study of the incidence of community-onset severe sepsis and septic shock in adults. All patients with weight and height documented in the medical records on admission were included.

**Results:**

The case fatality rate (CFR) was negatively correlating with increasing BMI. Outcomes included 28-day CFR (p-value = 0.002), hospital CFR (p-value = 0.039) and 1-year CFR (p-value<0.001). When BMI was applied as continuous variable in a multiple logistic regression together with other possible covariates, we still could discern that BMI was associated with decreasing 28-day CFR (OR = 0.93, 95% CI 0.88–0.98, p-value = 0.009) and 1-year CFR (OR = 0.94, 95% CI 0.91–0.97, p-value<0.001).

**Conclusion:**

The hypothesis and paradox of obesity being associated with higher survival rates in severe bacterial infections was confirmed in this prospective, population-based observational study.

## Introduction

Obesity is a rapidly growing global health concern with a significant negative impact on life expectancy and health economics [1, 2]. Compared to a population of normal weight, obese individuals are more prone to developing several forms of cancer and cardiovascular disease [3, 4]. Although the mechanisms that explain how obesity contributes to different diseases have not been completely elucidated, there is convincing evidence that obesity affects the immune system [5]. Obesity is claimed to be associated with immune cell dysfunction [5, 6] and an association between obesity and chronic low-grade inflammation has been demonstrated [7].

Although obesity has been demonstrated to have a significant impeding impact on the immune system, studies on how body mass index (BMI) impacts the outcomes of infections present diverging results. On the one hand, epidemiological studies indicate increased morbidity and mortality in obese individuals following COVID-19 and influenza infections as well as the requirement of longer antifungal treatment of candidemia and a higher risk of nosocomial infections post-surgery [8–13]. Furthermore, an association between BMI greater than 35 and severe Clostridioides difficile infection has been demonstrated [14]. On the other hand, epidemiological studies indicate an association between obesity and survival in community-acquired pneumonia [15–17]. Furthermore, several studies on how BMI affects the outcome of patients with septicemia and septic shock indicate equal or higher rates of survival among the obese compared to the non-obese [18–20]. These studies included mainly critically ill patients admitted to intensive care units. Exploring to what extent this obesity paradox generated in the studies from ICU cohorts and retrospective studies of specific diagnosis are applicable in a broader, more unselected cohort of patients treated for bacterial infections could have both prognostic and therapeutic implications. This prospective study aims to determine how BMI impacts the outcome of severe bacterial infections in a well-defined population-based cohort.

## Materials and methods

### Study cohort

This study is based on a cohort of patients included in a Swedish prospective, population-based, consecutive observational study of the incidence of community-onset severe sepsis and septic shock in adults [21]. Formal approval of the study was given by the Regional Ethics Committee in Gothenburg (no. 376–11.) In the study, all permanent adult residents admitted to a secondary-care hospital between September 2011 and June 2012 who started on intravenous antibiotic treatment within 48 hours based on the clinical suspicion of the bacterial infection, were evaluated [21]. Because of the observational nature of the study, the Regional Ethics Committee judged that no individual written informed consent was needed for cultures and biochemistry analyses that were included in the routine patient care nor for reviewing the patients’ electronic health record for the purpose of the study. All patients whose weight and height were documented on admission were included in this study. No additional biochemical analyses were performed, and no additional data were collected. All data were fully anonymized before access.

#### BMI classification

BMI was calculated as weight in kilograms divided by the square of the height in meters (kg/m^2^) and the patients were classified according to the World Health Organization’s (WHO) criteria: Underweight (BMI < 18.50 kg/m^2^), normal weight (BMI = 18.50 to 24.99 kg/m^2^), overweight (BMI = 25.0 to 29.99 kg/m^2^), obese (BMI = 30.0 to 34.99 kg/m^2^), and very obese (BMI > 35 kg/m^2^).

### Data collection

The following data was documented at baseline: age, gender, vital signs at admission, comorbid conditions (immunosuppressive disorders, cancer, liver failure, heart failure, chronic pulmonary disease, chronic renal failure, and diabetes), and laboratory data. During the hospital stay, the presence of bacteremia, site of infection, and microbiological data were registered. Regarding interventions, time to antibiotics and antibiotic therapy, in relation to preliminary diagnosis, were registered.

### Outcomes

The primary outcome was an all-cause 28-day case fatality rate (CFR), defined as death from any cause occurring within 28 days after admission. Secondary outcomes were in-hospital CFR, one-year CFR, defined as death from any cause occurring within a year after admission, ICU admission, and length of hospital stay, defined as the total number of days in the hospital from admission to discharge or death.

### Statistical analysis

Descriptive statistics are presented as frequencies and percentages for categorical variables and as a mean with standard deviation (SD) or a median with quartiles (Q_L_−Q_u_) for continuous data, depending on its distribution. To identify possible covariates, we used BMI, classified in five categories, and explored different variables and their distribution among the categories. The statistical tests used for comparisons between the categories were the Chi-square test for trends with categorical variables and the one-way analysis of variance (ANOVA) for trends with continuous variables, where normal distribution could be assumed, and the non-parametric Kruskal-Wallis test for variables where normal distribution could not be assumed.

To explore variables that could be predictors for survival, we used three binary outcome variables: 28-day CFR, in-hospital CFR, and one-year CFR. Three different logistic-regression models were generated. Relevant variables, based on the results in Table 1 (Demographic and clinical characteristics by BMI-group), were tested in a univariate-logistic-regression model. All statistically significant variables and tendencies (p-value < 0.2) were included in a multiple-logistic-regression model. The odds ratio (OR) is presented with 95% confidence intervals (CI). Missing value analyses were performed by exploring and comparing the group of patients where the BMI data was missing with the group where it was available. A method for “Multiple imputation of BMI” in cases where BMI was missing was applied. The purpose of multiple imputation is to generate possible values for missing values, creating several "complete" sets of data. Three different models were generated with multiple imputation, and a subsequent sensitivity analysis based on multiple-regression analyses were performed; p-values <0.05 were considered significant. All statistical analyses were performed with statistical package IBM SPSS v.25.

**Table 1:** Demographic and clinical characteristics by BMI-group.

	BMI	BMI	BMI	BMI	BMI	P-value^1^
	<18,5 (n = 54)	18,5–24,9 (n = 625)	25–29,9 (n = 602)	30–34,9 (n = 251)	>35 (n = 124)	
**Age, mean (SD)**	68 (22)	68 (20)	69 (16)	66 (16)	62 (14)	0.002*
**Female, n (%)**	33 (61.1)	278 (44.5)	237 (39.4)	123 (49.0)	65 (52.4)	0.507
**No comorbidity, n (%)**	16 (29.6)	191 (30.6)	170 (28.2)	64 (25.5)	24 (19.4)	0.011*
**Chronic cardiovascular disease, n (%)**	26 (48.1)	312 (49.9)	332 (55.1)	153 (61.0)	77 (62.1	<0.001*
**Chronic pulmonary disease, n (%)**	15 (27.8)	116 (18.6)	85 (14.1)	32 (12.7)	30 (24.2)	0.318
**Chronic renal failure, n (%)**	1 (1.9)	42 (6.7)	37 (6.1)	30 (12.0)	13 (10.5)	0.005*
**Chronic liver disease, n (%)**	0 (0)	3 (0.5)	3 (0.5)	1 (0.4)	2 (1.6)	0.259
**Diabetes, n (%)**	7 (13)	76 (12.2)	104 (17.3)	59 (23.5)	45 (36.3)	<0.001*
**Malignancy, n (%)**	9 (16.7)	133 (21.3)	117 (19.4)	30 (12.0)	8 (6.5)	<0.001*
**Immune suppression, n (%)**	4 (7.4)	39 (6.2)	31 (5.1)	15 (6.0)	5 (4.0)	0.340
**CRP, median (quartiles)**	89 (22–149)	104 (42–171)	100 (35–175)	101 (41–186)	114 (36–199)	0.286
**LPK, median (quartiles)**	12.6 (9.9–15.3)	11.9 (8.2–16.1)	11.5 (8.5–15.3)	12.2 (9.0–15.4)	11.8 (8.5–14.6)	0.942
**NLCR (neutrophile-to-lymphocyte-count ratio), median (quartiles)**	12.9 (7–24.5)	10.2 (5.9–17.3)	9.6 (4.9–16.6)	8.5 (5.7–15.3)	8.8 (4.9–16.2)	0.006*
**B-neutrophile**	11.0 (7.1–14.5)	9.2 (6.4–13.0)	9.1 (6.2–12.9)	9.5 (6.9–13.1)	9.1 (6.8–13.0)	0.952
**B-lymphocyte**	0.7 (0.5–1.2)	0.9 0.6–1.3)	1.0 (0.6–1.5)	1.1 (0.7–1.6)	1.0 (0.6–1.5)	<0.001
**Bacteremia, n (%)**	5 (9.3)	87 (13.9)	87 (14.5)	27 (10.8)	19 (15.3)	0.988
**Severe sepsis, n (%)**	12 (22.2)	121 (19.4)	104 (17.3)	44 (17.5)	25 (20.2)	0.612
**Therapy-related variables**						
**Time (hours) from arrival at hospital until antibiotic intervention, mean (SD)**	4.0 (7.0)	4.4 (7.9)	4.9 (7.5)	5.0 (7.8)	4.0 (6.0)	0.634
**Time (hours) from onset of disease until antibiotic intervention, median (quartiles)**	15 (6–35)	22 (8–54)	24 (8–57)	22 (8–55)	19 (8–53)	0.412
**Proportion of patients receiving adequate initial antibiotics, n (%)**	54 (100)	613 (98.4)	592 (98.3)	248 (98.8)	123 (99.2)	0.659
**Antibiotics, n (%)**						
**Penicillin G**	12 (22)	127 (20)	136 (23)	50 (20)	27 (22)	0.865
**Cefotaxime**	29 (54)	325 (52)	257 (43)	127 (51)	59 (48)	0.198
**Tobramycin**	14 (26)	135 (22)	139 (23)	52 (21)	23 (19)	0.401
**Cloxacillin**	0 (0)	21 (3)	18 (3)	6 (2)	11 (9)	0.036*
**Piperacillin/Tazobactam**	8 (15)	102 (16)	110 (18)	39 (15)	19 (15)	0.907
**Other**	6 (11)	62 (10)	90 (15)	33 (13)	9 (7)	0.030*

^1^ P-value for linear trend when BMI-class increases.

## Results

In this analysis of how BMI impacts the outcomes of bacterial infections, 75% of the patients from the trial (1656 of 2196) with registered length and weight were included. Compared to the excluded patients that did not have the registered values, there was no significant difference in gender representation or length of hospital stay. However, the included patients were significantly younger (67.9 vs. 71.4 years; p<0.001), had significantly higher co-morbidity (71.9 vs. 65.7%; p = 0.006) and significantly lower 28-day and one-year CFR (6.2 vs 15.9% and 19.8 vs. 31.5%, respectively; p<0.001), and a higher percentage of patients were admitted to the ICU (7.8 vs 4.4%, respectively; p = 0.008). The multiple-imputation method was used to replace missing values with BMI estimates, and a sensitivity analysis was performed. The results of the analysis are presented below.

The BMI profile of the study population was 3.3% underweight, 37.7% normal weight, 36.4% overweight, 15.2% obese, and 7.5% very obese. In Table 1, demographic characteristics and clinical data on the different categories are presented.

BMI significantly and inversely correlates with age (p = 0.002). No significant association between BMI and gender was identified, but an association was found between BMI and the presence of comorbid conditions (p = 0.011.) The proportion of patients with diabetes, chronic cardiovascular disease, and chronic renal failure increased significantly while the proportion of patients with malignancies diminished significantly with BMI (p<0.001, p<0.001, p = 0.005, and p<0.001, respectively). There was no significant difference in the prevalence of chronic liver or lung diseases or immunosuppressive disorders between the BMI groups.

Neither the proportion of patients with bacteremia nor those that fulfilled the criteria for severe sepsis differed significantly between the BMI groups. B-lymphocytes increased significantly with BMI (p<0.001), and a significant negative association was found between BMI and the neutrophile-to-lymphocyte-count ratio (NLCR) (p = 0.006). No significant difference was found in C-reactive protein (CRP) or leukocyte levels (LPK) as well as time-to antibiotic intervention from the onset of the disease or upon their arrival at the hospital. Moreover, over 98% of the patients in all BMI groups were judged to have received adequate empirical antibiotic treatment, i.e. treatment according to local guidelines or trailing resistance testing. The initial antibiotic therapy is presented in Table 1, and the presented drugs were either given alone or as a part of combination therapy.

Erysipelas, soft-tissue infection and diverticulitis diagnoses were more common as BMI increased while the frequency of pneumonia diagnosis was significantly lower with increasing BMI, as presented in Table 2.

**Table 2:** Prevalence of different diagnoses by BMI-group.

	BMI	BMI	BMI	BMI	BMI	P-value^1^
	<18,5 (n = 54)	18,5–24,9 (n = 625)	25–29,9 (n = 602)	30–34,9 (n = 251)	>35 (n = 124)	
**Diagnosis**						
**Pneumonia/Respiratory-tract infection, n (%)**	18 (33.3)	186 (29.8)	155 (25.7)	68 (27.1)	27 (21.8)	0.042
**Urinary-tract infection, n (%)**	7 (13.0)	98 (15.7)	86 (14.3)	36 (14.3)	12 (9.7)	0.189
**Erysipelas, n (%)**	0 (0)	13 (2.1)	29 (4.8)	17 (6.8)	18 (14.5)	<0.001
**Diverticulitis, n (%)**	0 (0)	11 (1.8)	25 (4.2)	12 (4.8)	5 (4.0)	0.008
**Soft-tissue infection, n (%)**	2 (3.7)	20 (3.2)	19 (3.2)	8 (3.2)	11 (8.9)	0.050

^1^ P-value for linear trend when BMI-class increases.

In a univariate analysis, the CFR diminished significantly with increasing BMI, as presented in Table 3. The identified negative correlation between BMI and CFR was significant for in-hospital (p-value = 0.039), 28-day (p-value = 0.002), and one-year CFR (p-value<0.001). The length of hospital and ICU stay as well as the proportion of patients admitted to the ICU did not differ between the BMI groups.

**Table 3:** Outcome variables by BMI-group.

	BMI	BMI	BMI	BMI	BMI	P-value^1^
	<18,5 (n = 54)	18,5–24,9 (n = 625)	25–29,9 (n = 602)	30–34,9 (n = 251)	>35 (n = 124)	
**In-hospital CFR, n (%)**	4 (7.4)	34 (5.4)	31 (5.2)	11 (4.4)	1 (1)	0.039*
**28-day CFR, n (%)**	5 (9.3)	48 (7.7)	36 (6.0)	12 (4.8)	1 (1)	0.002*
**One-year CFR, n (%)**	14 (26)	162 (26)	100 (17)	41 (16)	11 (9)	<0.001*
**Hospital stay (days), median (quartiles)**	8 (3–13)	5 (3–10)	5 (3–9)	5 (3–9)	5 (3–10)	0.135
**ICU admission, n (%)**	2 (4)	51 (8)	42 (7)	22 (9)	12 (10)	0.383
**Stay at ICU (days), median (quartiles)**	13 (5–21)	2 (1–3)	2 (1–4)	3 (1–6)	2 (1–4)	0.203

^1^ P-value for linear trend when BMI-class increases.

When BMI was included as a continuous variable in a multiple-logistic regression with other possible covariates, we could still discern that increasing BMI was associated with decreasing 28-day CFR (OR = 0.93; 95% CI 0.88–0.98; p-value = 0.009) and one-year CFR (OR = 0.94; 95% CI 0.91–0.97; p-value<0.001). The analysis also identified age and existing malignancy to be independent predictors for 28-day and one-year CFR. In addition, multiple-regression identified erysipelas and chronic renal failure as significant predictors for one-year CFR, as presented in Table 4.

**Table 4:** Risk factors for 28-day and one-year CFR. Univariate and multivariate logistic regression models.

	**28-day CFR**			
	**Univariate**		**Multivariate**	
**Variable**	**OR with 95% CI**	**P-value**	**OR with 95% CI**	**P-value**
**BMI**	0.94* (0.90–0.98)	0.003	0.93* (0.88–0.98)	0.009
**Age**	1.07* (1.06–1.09)	<0.001	1.06* (1.04–1.09)	<0.001
**Chronic cardiovascular disease**	2.33* (1.68–3.24)	<0.001	1.47 (0.85–2.56)	0.171
**Chronic renal failure**	1.41* (0.83–2.40)	<0.001	1.68 (0.82–3.45)	0.159
**Diabetes**	1.21 (0.83–1.77)	0.322	Not included	
**Malignancy**	1.86* (1.32–2.63)	<0.001	2.09* (1.28–3.43)	0.003
**NLCR**	1.01* (1.00–1.02)	0.038	1.00 (0.98–1.01)	0.996
**Erysipelas**	0.44 (0.16–1.22)	0.114	0.64 (0.15–2.75)	0.546
**Pneumonia/Respiratory-tract infection**	1.21 (0.88–1.67)	0.234	Not included	
**Diverticulitis**	0.97 (0.38–2.45)	0.949	Not included	
**Soft-tissue infection**	1.13 (0.54–2.38)	0.748	Not included	
	**One-year CFR**			
	**Univariate**		**Multivariate**	
**Variable**	OR with 95% CI	P-value	OR with 95% CI	P-value
**BMI**	0.94* (0.92–0.96)	<0.001	0.94* (0.91–0.97)	<0.001
**Age**	1.06* (1.05–1.07)	<0.001	1.06* (1.04–1.07)	<0.001
**Chronic cardiovascular disease**	2.16* (1.75–2.66)	<0.001	1.29 (0.92–1.81)	0.143
**Chronic renal failure**	2.26* (1.60–3.19)	<0.001	2.81* (1.76–2.50)	<0.001
**Diabetes**	1.37* (1.06–1.76)	0.015	1.17 (0.81–1.70)	0.396
**Malignancy**	3.84* (3.04–4.86)	<0.001	4.95* (3.60–6.81)	<0.001
**NLCR**	1.01* (1.006–1.02)	<0.001	1.00 (0.99–1.01)	0.316
**Erysipelas**	0.42* (0.22–0.79)	0.007	0.33* (0.12–0.93)	0.037
**Pneumonia/Respiratory- tract infection**	1.24 (0.99–1.53)	0.054	0.99 (0.73–1.36)	0.977
**Diverticulitis**	0.67 (0.34–1.34)	0.262	Not included	
**Soft-tissue infection**	0.56 (0.30–1.04)	0.065	1.45 (0.63–3.31)	0.380

Variables with p-value<0.2 from univariate analyses were included in the multivariate analysis.

### Sensitivity analysis

After multiple imputation when BMI value was missing, risk factors for 28-day and one-year CFR were identified. BMI was predicted and imputated based on covariates such as age, sex, weight, height, and comorbidity. Three different models based on the “multiple imputation” of BMI were generated and followed by multiple-regression analysis. These results confirm the results based on original data, as presented in Table 5.

**Table 5:** Multivariate logistic regression models after multiple imputation.

	**28-day CFR**					
	**Model 1**		**Model 2**		**Model 3**	
**Variable**	**OR with 95% CI**	**P-value**	**OR with 95% CI**	**P-value**	**OR with 95% CI**	**P-value**
BMI	0.95* (0.91–0.99)	0.022	0.96 (0.92–1.00)	0.050	0.95* (0.91–0.99)	0.023
Age	1.07* (1.06–1.09)	<0.001	1.07* (1.06–1.09)	<0.001	1.07* (1.06–1.09)	<0.001
Chronic cardiovascular disease	1.04 (0.70–1.54)	0.845	1.03 (0.70–1.53)	0.875	1.04 (0.70–1.54)	0.839
Chronic renal failure	1.35 (0.75–2.43)	0.323	1.32 (0.73–2.38)	0.357	1.35 (0.75–2.44)	0.316
Diabetes	/					
Malignancy	1.66* (1.12–2.45)	0.012	1.67* (1.13–2.47)	0.010	1.66* (1.12–2.46)	0.011
NLCR	1.00 (0.99–1.01)	0.369	1.00 (0.99–1.01)	0.348	1.00(0.99–1.01)	0.342
Erysipelas	0.52 (0.16–1.70)	0.279	0.51 (0.15–1.66)	0.262	0.52 (0.16–1.71)	0.283
Pneumonia/Respiratory infection	Not included					
Diverticulitis	Not included					
Soft-tissue infection	Not included					
	**One-year CFR**					
	**Multivariate model 1**		**Multivariate model 2**		**Multivariate model 3**	
**Variable**	**OR with 95% CI**	**P-value**	**OR with 95% CI**	**P-value**	**OR with 95% CI**	**P-value**
BMI	0.95* (0.92–0.97)	<0.001	0.95* (0.92–0.97)	<0.001	0.95* (0.92–0.97)	<0.001
Age	1.07* (1.05–1.08)	<0.001	1.07* (1.05–1.08)	<0.001	1.07* (1.05–1.08)	<0.001
Chronic cardiovascular disease	1.03 (0.79–1.36)	0.814	1.04 (0.79–1.36)	0.795	1.03 (0.78–1.36)	0.819
Chronic renal failure	2.32* (1.54–3.51)	<0.001	2.30* (1.52–3.47)	<0.001	2.33* (1.54–3.52)	<0.001
Diabetes	1.29 (0.95–1.76)	0.103	1.29 (0.95–1.76)	0.100	1.30 (0.96–1.77)	0.092
Malignancy	3.74* (2.85–4.91)	<0.001	3.75* (2.86–4.92)	<0.001	3.74* (2.85–4.91)	<0.001
NLCR	1.01* (1.00–1.02)	0.023	1.01* (1.00–1.02)	0.021	1.01* (1.00–1.02)	0.018
Erysipelas	0.55 (0.26–1.15)	0.113	0.54 (0.26–1.13)	0.104	0.55 (0.26–1.15)	0.114
Pneumonia/Respiratory tract infection	1.01* (0.78–1.30)	0.943	1.00 (0.77–1.29)	0.998	1.00 (0.78–1.29)	0.987
Diverticulitis	Not included					
Soft-tissue infection	0.90 (0.44–1.86)	0.782	0.92 (0.45–1.90)	0.831	0.89 (0.43–1.83)	0.750

Variables with p-value<0.2 from univariate analyses were included in the multivariate analysis.

## Discussion

We identified BMI as an independent predictor for the outcome of severe bacterial infections. Obese patients had a better prognosis than normal-weight and underweight patients. A key question is whether our results reflect a true benefit of being obese when affected by severe bacterial infections or if this can be explained with a confounder. We identified a negative correlation between survival and age, which is consistent with previous studies [20, 22]. BMI was associated with age—the obese were significantly younger, which is in line with two other studies [18, 20], though the association between BMI and survival remained when adjusted for age. Other identified variables that were associated with BMI in the study population, such as comorbid conditions and differences in the site of the infection, did not explain the relative benefit of being obese. Neither could we identify any differences in therapy that could explain an outcome related to BMI. Our results are restricted to initial antibiotic treatment and do not include changes in therapy, dosage, and duration that could explain differences in outcome. However, there is little support in previous research for a more optimized antibiotic treatment regime for the obese. Thus, while correlations between BMI and survival have been explained by co-variates in some studies [18, 22], we have identified no potential confounders. Moreover, our results are consistent with a large retrospective study by Prescott et al. where BMI was identified as an independent predictor of one-year survival in older Americans [20].

It is hard to explain why obesity is associated with higher survival rates in cases of severe bacterial infections but with higher mortality rates in cases of severe viral infections, such as influenza and COVID-19. It has been argued that survival in cases of severe infections depends on positive aspects of metabolic syndrome, such as energy reserves and an activated immune system [23, 24]. This is consistent with a greater chance of survival in cases of severe bacterial infections but is contradictory to the higher mortality rates in cases of severe viral infections. The alveolar hypoventilation that is common in obese patients could explain the worse outcome of lung infections related to influenza and COVID-19 in this group of patients but is not consistent with their greater chance of survival in cases of severe bacterial infections [25].

One hypothesis is that changes in the hormone and cytokine levels of the obese could be a reason behind the contradictory outcomes of different infections. The hyper-anabolic state of the obese, with an abundance of glucose and fatty acids, is associated with increased levels of leptin that stimulate the proliferation of adipocytes [7]. Moreover, the hyperplasia and hypertrophy of adipose tissue in the obese are associated with the increased necrosis of fragile adipocytes and changes in cytokine secretion that not only result in the increased recruitment of immune cells but also affect how immune cells operate [6, 7, 26]. The development of proinflammatory T-cell subtypes such as Th1 and Th17 is stimulated by leptin, which is enhanced in the obese [27, 28]. However, whether an obesity-induced change in T-cell population can explain the contradictory impact of BMI on the outcomes of different infections needs to be further investigated.

Another important aspect is to what extent the study population is representative of society and how it differs from other studied populations. In this study, 23% were obese or very obese whereas 13% of the national population was reported to be obese or very obese in a yearly national report from the Public Health Agency of Sweden [29]. The larger proportion of obese or very obese individuals in this study could signal that the incidence of severe infections is higher in their case. However, research indicates that self-reported data, as in the national report, could underestimate BMI [30]. The proportion of the obese and very obese in this study is similar to, or somewhat lower than, studies with sepsis as an outcome, which spans between 23 and 35% [18, 20, 31].

Moreover, compared to cohorts with the diagnosis of sepsis, overall CFR was lower in this study—8.6% for 28-day and 22.7% for one-year CFR [18, 20, 31]. A plausible explanation of the lower CFR is that the community-based population consists of a broader spectrum of patients and includes a larger proportion of less-critically-ill patients compared to the ICU cohorts with severe sepsis or septic shock. However, no differences were found that could signal differences in disease severity in different BMI groups, such as ICU admittance or severe sepsis; neither did the sites of infection in this study’s cohort differ from other studies where the site of infection was registered. An increasing BMI was associated with a higher rate of skin and soft-tissue infections and a lower rate of pneumonia, consistent with findings in a cohort of septic-shock patients [18]. The identified independent association between one-year survival and erysipelas in this study is interesting, though it could not be explained with the collected data. One speculation that needs to be further studied is that the long-term prophylactic antibiotic therapy that erysipelas patients sometimes receive contributes to their chance of survival.

In contrast to previous studies that indicate that obesity-related inflammation is linked to elevated levels of neutrophils and lymphocytes, this study of patients with ongoing bacterial infections identifies a significant association of BMI with lymphocytes but not with neutrophils; this is illustrated by a negative association between the neutrophil-to-lymphocyte-count ratio and BMI [32]. Why BMI is associated with lymphocytes in this cohort has no obvious explanation but warrants further studies.

This study has weaknesses. One is that 25% of the patients included in the original study did not have their BMI data registered. In two other large studies, over 95% of the included patients had both weight and height registered [20, 31]. Still, a meticulous sensitivity analysis indicates that our results are robust. Another weakness is that this was a single-center study, which makes the results less generalizable than a multi-center study. However, to our knowledge this is the first consecutive observational study that evaluates the outcome of severe bacterial infections in relation to BMI in a population-based cohort. By studying the prognosis in a population-based cohort, rather than ICU-based cohorts, selection bias, based on potential ICU thresholds such as age and comorbidities, is diminished. Importantly, the study’s design makes the results relevant for physicians meeting unselected patients at emergency departments in clinical practice. As Huttunen discuss in a review on obesity and infection [33], there is still a lack of data on both the risk of infection and the outcome of infections in obese patients. Our study helps to close this knowledge gap.

### Conclusion

This study indicates that the obesity survival paradox is relevant in a community-based cohort. Overall, our results identify a relative benefit of higher BMI in cases of severe bacterial infections but also indicate higher incidence. With the spread of obesity, more knowledge of how BMI impacts the incidence, presentation, and outcome of different types of severe infections is vital. Further population-based studies are warranted.
